# Identification of the Cancer Cell Proliferation and Survival Functions of proHB-EGF by Using an Anti-HB-EGF Antibody

**DOI:** 10.1371/journal.pone.0054509

**Published:** 2013-01-21

**Authors:** Shuji Sato, Hiroko Kamada, Takahiro Watanabe, Isamu Tsuji, Jinhong Fan

**Affiliations:** 1 Takeda San Francisco, Inc., South San Francisco, California, United States of America; 2 Pharmaceutical Research Division, Takeda Pharmaceutical Company Limited, Fujisawa, Kanagawa, Japan; University of Torino, Italy

## Abstract

**Purpose:**

Heparin-binding epidermal growth factor-like growth factor (HB-EGF) is a member of the epidermal growth factor family. The membrane-bound proHB-EGF is known to be a precursor of the soluble form of HB-EGF (sHB-EGF), which promotes cell proliferation and survival. While the functions of sHB-EGF have been extensively studied, it is not yet fully understood if proHB-EGF is also involved in cellular signaling events. In this study, we utilized the anti-HB-EGF monoclonal antibodies Y-142 and Y-073, which have differential specificities toward proHB-EGF, in order to elucidate proHB-EGF functions in cancer cells.

**Experimental Design:**

The biological activities of proHB-EGF were assessed in cell proliferation, caspase activation, and juxtacrine activity assays by using a 3D spheroid culture of NUGC-3 cells.

**Results:**

Y-142 and Y-073 exhibited similar binding and neutralizing activities for sHB-EGF. However, only Y-142 bound to proHB-EGF. We could detect the function of endogenously expressed proHB-EGF in a 3D spheroid culture. Blocking proHB-EGF with Y-142 reduced spheroid formation, suppressed cell proliferation, and increased caspase activation in the 3D spheroid culture of NUGC-3 cells.

**Conclusions:**

Our results show that proHB-EGF acts as a cell proliferation and cell survival factor in cancer cells. The results suggest that proHB-EGF may play an important role in tumor progression.

## Introduction

HB-EGF is a member of the epidermal growth factor (EGF) family of growth factors [1]. It is synthesized as a transmembrane protein, proHB-EGF, composed of a signal peptide; a pro-peptide; and heparin-binding, EGF-like, juxtamembrane, transmembrane, and cytoplasmic domains [2]. During cellular stress, proHB-EGF undergoes ectodomain shedding that releases the soluble form, sHB-EGF, and the intracellular C-terminal fragment (CTF) [3], [4]. sHB-EGF exerts a potent mitogenic and/or chemotactic activity through the activation of its receptors EGFR and ERBB4 [1], [5], [6]. The CTF translocates into the nucleus and induces the gene expression of cyclinA and cyclinD2 by suppressing the function of PLZF and Bcl6, respectively [7], [8].

In addition to being a precursor of sHB-EGF and CTF, proHB-EGF has unique properties as a diphtheria toxin receptor [9], a cell adhesion molecule [10], and a juxtacrine factor [11]. Diphtheria toxin binding to proHB-EGF is potentiated by CD9 or heparin-like molecules [12], [13], and the binding causes the inhibition of protein synthesis through the internalization of the diphtheria toxin-proHB-EGF complex. As a cell adhesion molecule, proHB-EGF contributes to blastocyst adhesion to the uterus during implantation in mice [10]. The juxtacrine activity of proHB-EGF was first noted in a coculture system, where proHB-EGF-overexpressing cells were seeded on EGFR-overexpressing cells [11]. To isolate and assess the signaling initiated by proHB-EGF separately from that initiated by sHB-EGF, the proHB-EGF-overexpressing cells were fixed with formalin, thereby preventing the release of sHB-EGF. In this coculture system, the proHB-EGF-overexpressing cells promoted DNA synthesis and prevented apoptosis in the EGFR-overexpressing cells in some of the studies where it was used [11], [14], [15]. In contrast, when the intact proHB-EGF-overexpressing cells were not fixed with formalin, they inhibited DNA synthesis and promoted apoptosis in the EGFR-overexpressing cells in a modified coculture condition [16]. The functions of proHB-EGF were also evaluated by analyzing the effects of proHB-EGF overexpression on autonomous cellular events. The proHB-EGF overexpression suppressed or promoted cell proliferation in different cell lines [17], [18]. Thus, the roles of proHB-EGF have not been consistently or clearly elucidated.

In this study, we have assessed the functions of proHB-EGF in cancer cells by using 2 anti-HB-EGF monoclonal antibodies that have different specificities toward proHB-EGF. Our findings suggest that proHB-EGF plays roles in the proliferation and survival of cancer cells.

## Materials and Methods

### Materials

The anti-HB-EGF monoclonal antibodies Y-073 and Y-142 and sHB-EGF were previously generated [19]. In brief, Y-142 was prepared by immunizing BALB/c mice (Japan Clea) with subcutaneous injections of keyhole limpet hemocyanin-conjugated sHB-EGF and abdominal injections of 293F cells (Invitrogen) transiently transfected with a proHB-EGF expression plasmid. Y-073 was obtained by immunizing BALB/c mice with subcutaneous injections of keyhole limpet hemocyanin-conjugated sHB-EGF. Both antibodies were purified from their hybridoma culture supernatant with rProteinA Sepharose (GE Healthcare). sHB-EGF was prepared from the culture supernatant of 293F cells (Invitrogen) transfected with a sHB-EGF expression plasmid [19]. We also used the following reagents: mouse control IgG and horseradish peroxidase-labeled (HRP-labeled) anti-mouse IgG antibody from Jackson ImmunoResearch Laboratories; Alexa488-labeled anti-mouse IgG antibody, HRP-labeled anti-goat IgG antibody, and HRP-labeled anti-rabbit IgG antibody from Invitrogen; anti-amphiregulin (anti-ARG) monoclonal antibody, anti-HB-EGF polyclonal antibody, anti-EGFR polyclonal antibody, and biotinylated anti-EGFR polyclonal antibody from R&D Systems; anti-β-actin antibody from Cell Signaling Technology; erlotinib from Selleck Chemicals; biotinylated anti-phosphotyrosine antibody from Millipore; sulfotagged streptavidin from Meso Scale Discovery; and phorbol 12-myristate 13-acetate (PMA) from Wako.

### Cell Culture

NUGC-3 stomach cancer cells (Japanese Collection of Research Bioresources), 5637 bladder cancer cells (American Type Culture Collection), and BxPC-3 pancreatic cancer cells (American Type Culture Collection) were maintained in RPMI1640 medium supplemented with 10% serum. EFO-27 ovarian cancer cells (DMSZ) were maintained in RPMI1640 medium with 20% serum. The cells were maintained in 2D cell culture plates, and for 3D spheroid culture experiments, they were transferred to a Celltight Spheroid culture plate (Sumitomo Bakelite).

### Flow Cytometry

Cells were detached from a culture dish with cell dissociation buffer (Invitrogen) and incubated with anti-HB-EGF monoclonal antibody or anti-ARG monoclonal antibody for 1 h at 4°C. After washing with PBS containing 1% bovine serum albumin (BSA), the cells were incubated with Alexa488-labeled secondary antibody for 1 h at 4°C. Protein expression was measured using a FACS CantoII instrument (Becton Dickinson), and the data were then analyzed with the FlowJo software (Tree Star). To generate a positive control for the binding of the anti-ARG antibody, EFO-27 cells were transfected with an ARG expression plasmid by using Lipofectamine 2000 (Invitrogen).

### sHB-EGF-binding Assay

The binding activity of anti-HB-EGF monoclonal antibody to sHB-EGF was detected as previously described [19]. sHB-EGF or BSA was immobilized at 1 µg/mL on a 96-well plate overnight at 4°C. After nonspecific binding was blocked with PBS containing 1% BSA, anti-HB-EGF monoclonal antibody was incubated at various concentrations in the plate. HRP-labeled anti-mouse IgG antibody was then reacted for 1 h at room temperature. TMB Peroxidase EIA Substrate (Bio-Rad) was then added into each well. Antibody binding to sHB-EGF was detected by measuring the absorbance at 450 nm by using a SPECTRA MAX plate reader (Molecular Devices).

### EGFR Phosphorylation Assay

The inhibitory activity of anti-HB-EGF monoclonal antibody against sHB-EGF-induced EGFR phosphorylation was detected as previously described [19]. NUGC-3 cells were seeded at 1 × 10^4^ cells/well in RPMI1640 medium with 1% serum. After a 1-d incubation period, the cells were treated with various concentrations of anti-HB-EGF antibody and 10 nM sHB-EGF for 15 min at 37°C. Cell lysates were prepared with cell lysis buffer (Cell Signaling Technology) containing a protease inhibitor cocktail (Roche Applied Science) and a phosphatase inhibitor cocktail (Sigma Aldrich) and were used for analysis with a pEGFR detection ELISA kit (R&D Systems).

### Cell Proliferation and Caspase Assays

Cells were seeded at a concentration of 1 × 10^4^ cells/well in RPMI1640 medium containing 1% serum into Celltight Spheroid culture plates. We measured caspase 3/7 activation after a 1-d incubation period by using Caspase-Glo 3/7 (Promega) and measured cell proliferation after a 3-d incubation period by using CellTiter-Glo (Promega). Measurements for both parameters were obtained using an Envision instrument (Perkin Elmer). Cell proliferation was calculated as the percentage of the proliferation level measured in the absence of antibody.

### siRNA Transfection

NUGC-3 cells were transfected with negative control siRNA or HB-EGF siRNA (Dharmacon) by using Lipofectamine 2000 during 2D cell culture. The HB-EGF siRNA used in this study was a mixture of 4 siRNAs, purchased from Dharmacon (cat. L-019624-00-005) and was transfected at a final concentration of 6 nM (1.5 nM for each siRNA). After transfection, the cells were transferred to a 3D spheroid culture. siRNA-transfected cells were then used in the cell proliferation assay. Reduction in HB-EGF protein expression was examined by western blot. HB-EGF expression was detected by anti-HB-EGF polyclonal antibody and HRP-labeled anti-goat IgG antibody. β-actin, an internal loading control, was detected with anti-β-actin antibody and HRP-labeled anti-rabbit IgG antibody.

### Measurement of proHB-EGF Juxtacrine Activities

NUCG-3 cells were incubated with 200 nM Y-142 or Y-073 for the indicated times under the spheroid culture condition. Mouse control IgG (200 nM) and 1 nM erlotinib were also used in the assay as negative and positive controls, respectively. For the detection of EGFR phosphorylation, cell lysates prepared as described above were incubated in a Sector Imager 6000 Reader plate (Meso Scale Discovery) precoated with anti-EGFR polyclonal antibody. The plate was then incubated with biotinylated anti-phosphotyrosine antibody, followed by incubation with sulfotagged streptavidin. For the detection of total EGFR, the cell lysates were incubated in a Sector Imager 6000 Reader plate coated with anti-EGFR polyclonal antibody. Biotinylated anti-EGFR polyclonal antibody was then incubated in the plate, followed by incubation with sulfotagged streptavidin. Read T buffer (Meso Scale Discovery) was then added, and chemiluminescence was measured with a Sector Imager 6000 instrument (Meso Scale Discovery). The EGFR phosphorylation signal was normalized to the total EGFR signal and then calculated as the percentage of the maximum EGFR phosphorylation that was measured in the absence of Y-142, Y-073, control IgG, and erlotinib.

For the detection of the phosphorylation of ERK1/2 and AKT, we used the phosphoERK1/2 and phosphoAKT kits (Meso Scale Discovery), respectively, according to the supplier’s instructions. In brief, cell lysates prepared as described above were added to the plate with spots of anti-ERK1/2 and anti-phosphoERK1/2 antibodies, or with spots of anti-AKT and anti-phosphoAKT antibodies. Then, each plate was incubated with sulfotagged anti-ERK1/2 antibody or with sulfotagged anti-AKT antibody. Read T buffer with surfactant was then added, and chemiluminescence was measured with a Sector Imager 6000 instrument. The ERK1/2 or AKT phosphorylation signal was normalized to the total ERK1/2 or AKT signal, respectively. ERK1/2 or AKT phosphorylation was then calculated as the percentage of the maximum ERK1/2 or AKT phosphorylation, respectively, which was measured in the absence of Y-142, Y-073, control IgG, and erlotinib.

### Detection of CTF

NUGC-3 cells were treated with 200 nM Y-142 for 3 d or 50 nM PMA for 30 min as a control for ectodomain shedding induction and CTF production. Cell lysates prepared as described above were subjected to western blot. The CTF fragment was detected using the anti-CTF polyclonal antibody and HRP-labeled anti-rabbit IgG antibody. Anti-CTF polyclonal antibody was generated by immunizing a rabbit with an antigen peptide corresponding to amino acids 185–208 of proHB-EGF, as described previously [20].

## Results

### Characterization of Y-142 and Y-073

**Figure 1:**
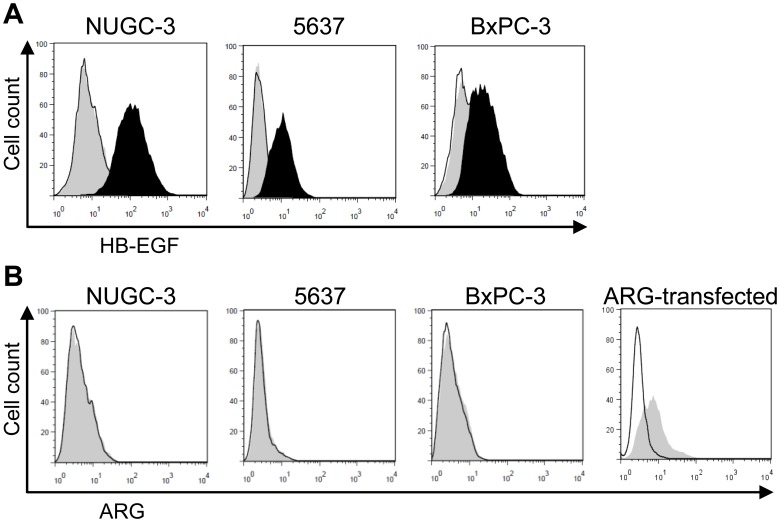
Comparison of Y-142 and Y-073 binding activities toward proHB-EGF. A. Binding of anti-HB-EGF antibody to proHB-EGF. The cells were incubated with anti-HB-EGF antibody, followed by incubation with Alexa488-labeled anti-mouse IgG antibody. The binding was detected by flow cytometry. No IgG, black line; Y-142, black histogram; Y-073, gray histogram. B. Expression of amphiregulin (ARG) in cancer cell lines. Cells were incubated with anti-ARG antibody, followed by incubation with Alexa488-labeled anti-mouse IgG antibody. The binding activity of anti-ARG antibody was confirmed using EFO-27 cells transfected with an ARG expression plasmid. No IgG, black line; anti-ARG antibody, gray histogram.

A large number of neutralizing anti-HB-EGF antibodies were generated using a hybridoma approach as reported previously [19]. During the characterization of the neutralizing antibodies, we found that the antibodies showed differential binding toward the transiently overexpressed membrane-bound form of HB-EGF, proHB-EGF. This finding allowed us to hypothesize that characterization of the antibodies would lead to the identification of proHB-EGF function. We selected 2 antibodies, Y-142 as a proHB-EGF binder and Y-073 as a non-proHB-EGF binder, and compared their characteristics in cancer cells. First, we confirmed that Y-142 and Y-073 had different binding profiles to cancer cell lines endogenously expressing proHB-EGF. Y-142 bound to the tested cancer cell lines, whereas Y-073 showed no binding (Fig. 1A).

EGFR ligands include HB-EGF, amphiregulin (ARG), tumor growth factor α, neuregulin β1, EGF, and betacellulin, and our previous study showed that Y-142 recognizesARG in addition to HB-EGF [21]. Both ligands are expressed as membrane-bound forms on the cell surface [22]. Therefore, to confirm that the Y-142 binding to cells was attributable to its recognition of proHB-EGF, and not to ARG, ARG expression was examined by flow cytometry, and no ARG expression was detected (Fig. 1B). To rule out the possibility that the anti-ARG antibody did not have binding activity for ARG, we used transfected ARG as a positive control. This result further confirmed that the binding of Y-142 to the tested cancer cell lines was attributable to its recognition of proHB-EGF. We also tested the EGFR ligand specificity of Y-073, and Y-073 specifically bound to HB-EGF (data not shown).

**Figure 2:**
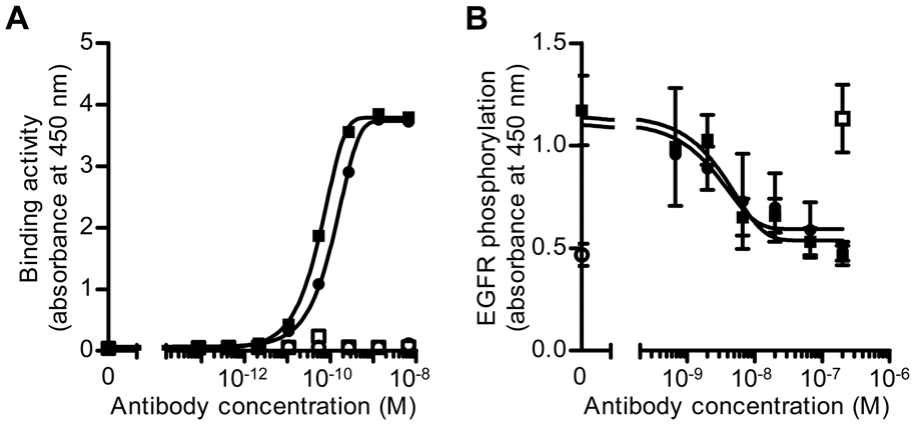
Comparison of Y-142 and Y-073 biological activities toward sHB-EGF. A. Binding activity of Y-142 and Y-073 toward sHB-EGF. Anti-HB-EGF antibody was incubated at various concentrations in an sHB-EGF-coated or a BSA-coated plate. Antibody binding was detected with an HRP-labeled anti-mouse IgG antibody. Y-142 binding to BSA, white square; Y-073 binding to BSA, white circle; Y-142 binding to sHB-EGF, black square; Y-073 binding to sHB-EGF, black circle. The data points represent the mean ± standard deviation (SD) of values acquired in duplicate. B. Neutralizing activity of Y-142 and Y-073 against sHB-EGF-induced EGFR phosphorylation. Anti-HB-EGF antibody was incubated at the indicated concentrations in the presence of 10 nM sHB-EGF with NUGC-3 cells for 15 minutes at 37°C. Cell lysates were incubated on an anti-EGFR antibody-coated plate, followed by incubation with HRP-labeled anti-phosphotyrosine antibody. No sHB-EGF, white circle; sHB-EGF and control IgG, white square; sHB-EGF and Y-142, black square; sHB-EGF and Y-073, black circle. The data points represent the mean ± SD of values acquired in triplicate.

To further examine the characteristics of the antibodies, we tested their binding activity to sHB-EGF by ELISA. Interestingly, Y-142 and Y-073 bound to sHB-EGF with comparable EC_50_ values of 56 pM and 110 pM, respectively, and neither of them bound to the negative control BSA (Fig. 2A). In addition, we tested their neutralizing activity on sHB-EGF-induced EGFR phosphorylation. The NUGC-3 stomach cancer cell line was used to detect EGFR phosphorylation because of its high EGFR expression. As shown in Fig. 2B, both of the antibodies inhibited the phosphorylation of EGFR in a concentration-dependent manner with similar IC_50_ values (Y-142 = 3.8 nM; Y-073 = 4.1 nM). These findings indicated that only Y-142 had the ability to bind to proHB-EGF, although both Y-142 and Y-073 had similar binding and neutralizing activities toward sHB-EGF.

### Cell Proliferation and Survival Functions of proHB-EGF

**Figure 3:**
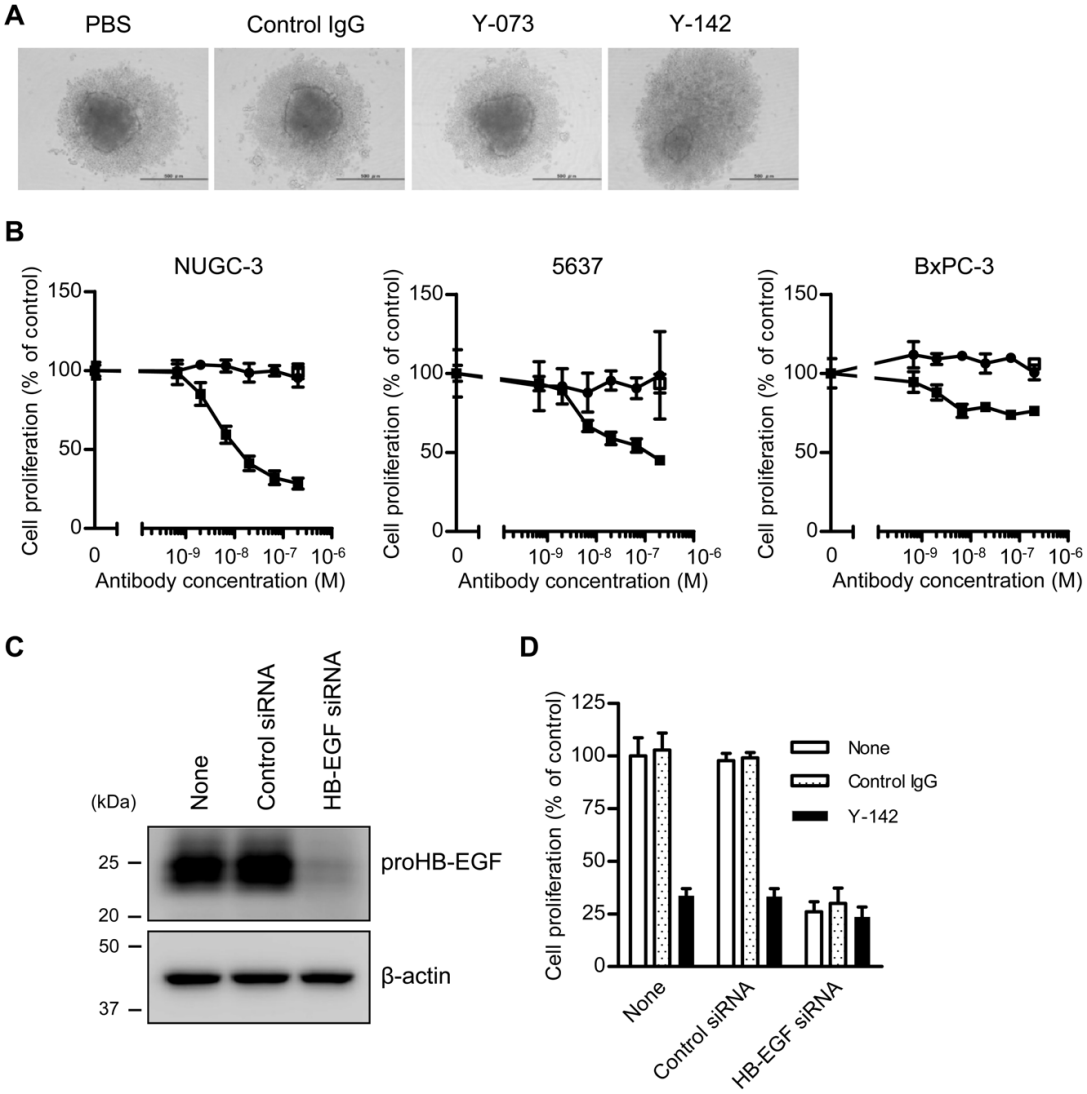
Spheroid-forming and cell-proliferation effects of proHB-EGF. Spheroid formation and cell proliferation by proHB-EGF function were assessed under Y-142 treatment. The cells were seeded into a spheroid culture plate in the presence of 1% serum. After a 1-d incubation period, the cells were treated with the indicated concentrations of anti-HB-EGF antibody and cultured for 3 d. A. Spheroid formation of NUGC-3 cells. The bar in each image indicates 500 µm. B. Inhibition of proHB-EGF-dependent cell proliferation by Y-142. Cell proliferation was measured by CellTiter-Glo and is shown as the percentage of the proliferation of PBS-treated cells. Control IgG, white square; Y-142, black square; Y-073, black circle. The data points represent the mean ± SD of values acquired in triplicate. C. Reduction in HB-EGF protein by HB-EGF siRNA. Cell lysates of siRNA-transfected NUGC-3 cells were subjected to SDS-PAGE. HB-EGF was detected by western blotting, with β-actin as the internal control. D. Confirmation of proHB-EGF-dependent cell proliferation by HB-EGF siRNA. NUGC-3 cells were transfected with HB-EGF siRNA prior to spheroid culture. One day after the siRNA transfection, the cells were seeded into a spheroid culture plate. Cell proliferation was measured by CellTiter-Glo and shown as a percentage of the cell proliferation of PBS-treated cells. The data points represent the mean ± SD of values acquired in triplicate.

A previous study has shown that proHB-EGF is involved in cell-cell contact [10]. In a 3D spheroid culture, cancer cells undergo more extensive cell-cell contacts than in a 2D culture [23]. We speculated that the cell-cell contact-mediated proHB-EGF effect might be more pronounced and easier to detect in the 3D spheroid culture. To this end, we established a 3D spheroid culture cell-based assay by using NUGC-3 cells. When NUGC-3 cells were seeded in a spheroid culture plate, they formed a spheroid with a small cell mass in the center core and loosely distributed cells surrounding the core (dark gray and light gray, respectively, in the PBS control in Fig. 3A). Interestingly, under the Y-142 treatment, the cells were more diffuse and had a smaller center core and a larger halo. The morphological change caused specifically by Y-142 suggested that this change in spheroid morphology was attributable to the modulation of proHB-EGF functions. We further explored the proHB-EGF functions by using the 3D spheroid culture assay. In the cell proliferation assay, Y-142 significantly inhibited the proliferation of NUGC-3 in a concentration-dependent manner (Fig. 3B). The inhibition of cell proliferation reached 72% at the maximum antibody concentration. In contrast, Y-073 had no effect on proliferation. Similar results were obtained in the other cell lines tested, that is, 5637 and BxPC-3 (Fig. 3B). Since Y-073 inhibited the functions of sHB-EGF and had no effect on any of the proHB-EGF cell lines in the spheroid culture, it was suggested that the function of endogenous proHB-EGF was dominant in the spheroid culture, compared to the function of endogenous sHB-EGF. These results indicated that proHB-EGF acted as an important cell proliferation factor in cancer cells.

To further confirm that the inhibition of cell proliferation was caused by the perturbation of proHB-EGF function, we evaluated the effect of HB-EGF siRNA on cell proliferation in the spheroid assay for NUGC-3 cells. Reduced expression of proHB-EGF due to HB-EGF siRNA was confirmed by western blotting (Fig. 3C). In accordance with the knockdown of proHB-EGF expression, HB-EGF siRNA caused reduced cell proliferation (Fig. 3D). We found that the extent of inhibition of cell proliferation by HB-EGF siRNA was similar to that observed with Y-142. These results are consistent with the notion that Y-142 inhibits proHB-EGF function, which may lead to reduced cell proliferation.

**Figure 4:**
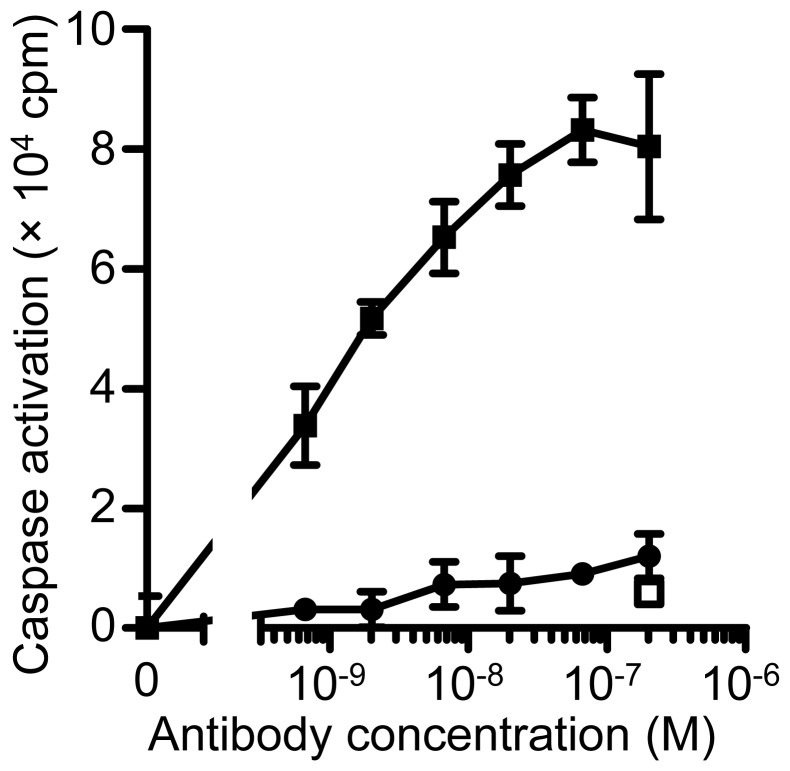
Cell survival signaling through proHB-EGF. The cell survival activity of proHB-EGF was tested by measuring caspase activation. NUGC-3 cells were seeded into a spheroid culture plate in the presence of 1% serum. After a 1-d incubation period, the cells were treated with Y-142 and cultured for 1 d. Caspase activation was measured by Caspase-Glo 3/7. Control IgG, white square; Y-142, black square; Y-073, black circle. The data points represent the mean ± SD of values acquired in triplicate.

To explore the molecular mechanism underlying the functions of proHB-EGF in cell survival, we measured caspase activation, a key event in apoptosis [24], in NUGC-3 cells. As indicated in Fig. 4, Y-142 promoted the activation of caspase-3/7 (Fig. 4), while Y-073 had no effect. This result indicated that proHB-EGF has cell survival activity in cancer cells and that inhibiting proHB-EGF triggers caspase-mediated apoptosis.

### Inhibition of proHB-EGF Juxtacrine Activities by Y-142

**Figure 5:**
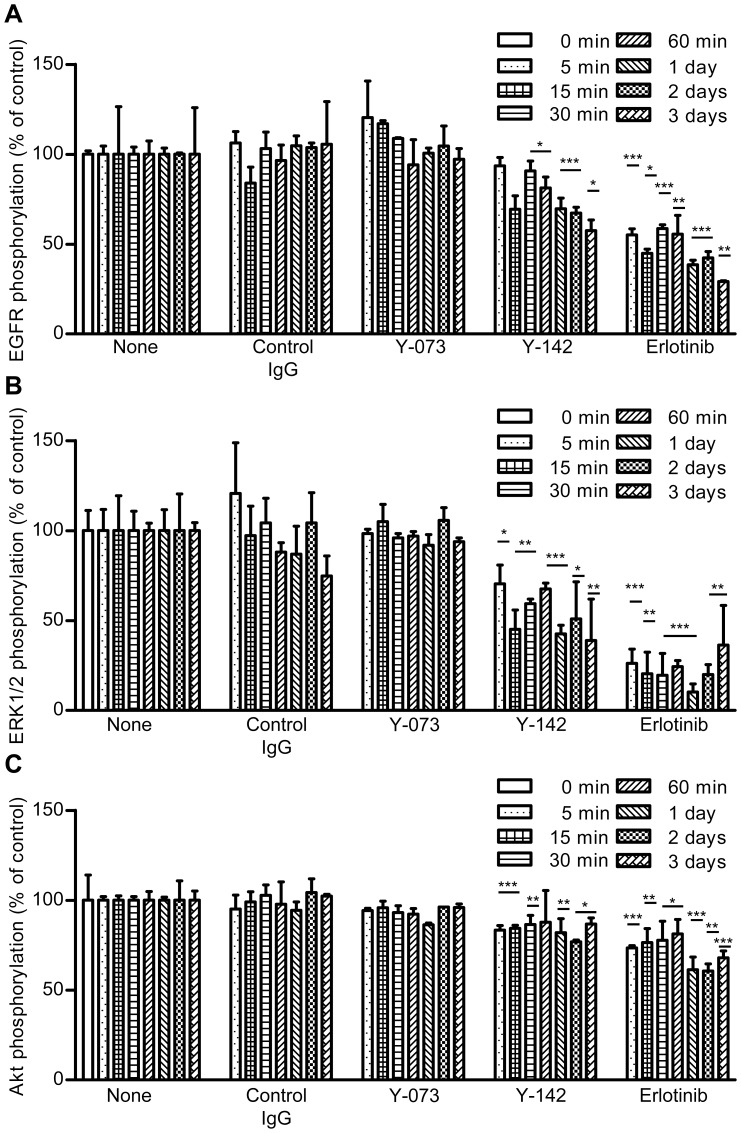
Inhibition of proHB-EGF juxtacrine activity by Y-142. proHB-EGF juxtacrine activity was measured using Y-142. NUGC-3 cells in a spheroid culture plate were treated with 200 nM control IgG, 200 nM Y-142, 200 nM Y-073, or 1 nM erlotinib for the indicated times. A. Phosphorylation of EGFR. B. Phosphorylation of ERK1/2. C. Phosphorylation of AKT. Phosphorylation was calculated as the percentage of the phosphorylation of PBS-treated cells. The data points represent the mean ± SD of values acquired in triplicate. Statistical analysis was performed using the unpaired t-test (one-tailed, vs. PBS-treated sample at the corresponding time point). *, p<0.05; **, p<0.005; ***, p<0.0005.

Unlike sHB-EGF, which acts by autocrine and paracrine mechanisms, juxtacrine signaling is a mechanism unique to proHB-EGF [11]. We hypothesized that the effect of Y-142 may be mediated specifically through the inhibition of proHB-EGF juxtacrine signaling. Because the juxtacrine activity was reported to be EGFR-mediated [11], we measured the phosphorylation of EGFR as well as of its downstream proteins ERK1/2 and AKT, which are involved in cell proliferation and caspase-mediated apoptosis, respectively, in NUGC-3 cells. The EGFR inhibitor erlotinib, used as a positive control, inhibited the phosphorylation of EGFR, ERK1/2, and AKT (Fig. 5). Similar to the effect of erlotinib, Y-142 decreased the phosphorylation of EGFR, ERK1/2, and AKT, whereas Y-073 as well as control IgG did not. The inhibition of their phosphorylation by Y-142 was significant at 15 min after the treatment and lasted for up to 3 d, suggesting that the juxtacrine signal was constitutively switched on in the spheroid. These data suggested that proHB-EGF juxtacrine activity leads to cell proliferation as well as cell survival activity.

### No Effect of Y-142 on CTF Generation

**Figure 6:**
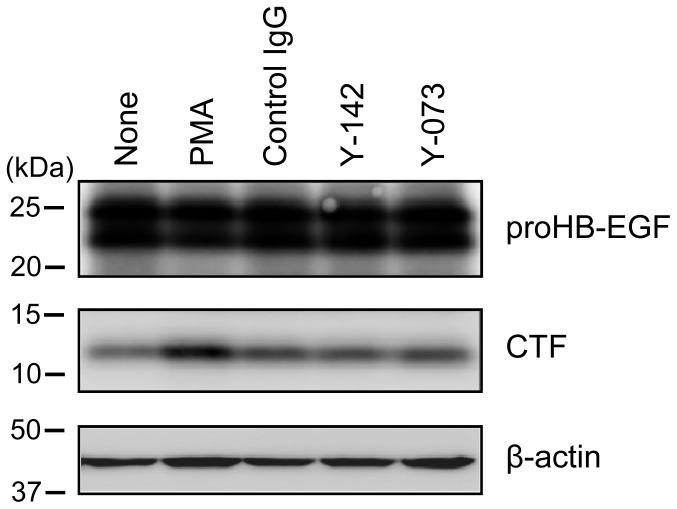
Y-142 did not affect CTF production. The effect of CTF on the observed inhibition of cell proliferation (Fig. 3B) was examined by measuring the amount of CTF. Cell lysates of the PMA-, control IgG, Y-142-, or Y-073-treated NUGC-3 cells were subjected to SDS-PAGE. CTF was detected with anti-CTF polyclonal antibody in western blotting.

CTF, which is produced along with sHB-EGF by ectodomain shedding, drives cell proliferation [25]. To evaluate the contribution of CTF in the inhibition of cell proliferation by Y-142, the CTF amount was examined in NUGC-3 cells. PMA [26] was used as controls to stimulate the ectodomain shedding and CTF production. As shown in Fig. 6, the amount of CTF generated in the presence and absence of Y-142 was not substantially different, suggesting that Y-142 had no impact on the generation of CTF.

## Discussion

HB-EGF consists of a membrane-bound form (proHB-EGF), a soluble form (sHB-EGF), and a C-terminal fragment (CTF), and each form has a unique biological activity. Among these 3 forms, the function of sHB-EGF has been widely evaluated using recombinant proteins. To date, the biological activity of proHB-EGF has been suggested on the basis of the effect of transfected proHB-EGF on the proliferation and survival of EGFR-expressing cells [11], [14]–[16] or on the basis of the effect of proHB-EGF overexpression on autonomous cell proliferation [17], [18]. However, information on proHB-EGF function has been scanty and inconsistent, possibly because of the lack of a method for specifically activating or inhibiting proHB-EGF. In some other studies, uncleavable mutant proHB-EGF (uc-proHB-EGF), which has 2 amino acid replacements (Leu148 and Pro149) in the cleavage site, was used to evaluate proHB-EGF function [26]. uc-proHB-EGF has been shown to possess juxtacrine activity in formalin-fixed EGFR-expressing cells [27]. If uc-proHB-EGF fully represents wild type proHB-EGF functions even in live cells, this mutant might be useful in identifying proHB-EGF functions in spheroid culture. Recently, another version of uc-proHB-EGF, whose cleavage site was deleted, was used to study proHB-EGF functions. This uc-proHB-EGF prevented anoikis of Madin-Darby canine kidney cells [28]. However, in comparison with these approaches used so far, there are some advantages in our approach using a 3D spheroid culture and a functional anti-HB-EGF antibody: the functions of endogenously expressed proHB-EGF can be detected, and formalin fixation, which may cause artifacts, is not required because there is no endogenous sHB-EGF function.

Accumulated reports showed that spheroid culture mimics the cancer environment better than a 2D culture in terms of cell-cell contacts, drug resistance, drug penetration, and nutrient efficiency [23], [29]; therefore, the functions of endogenously expressed proHB-EGF found in the 3D spheroid suggest that proHB-EGF may play important roles in tumor progression. In this study, we used 3 cancer cell lines endogenously expressing proHB-EGF. Y-142 inhibited the proliferation of NUGC-3 cells by 72%, 5637 cells by 55%, and BxPC-3 cells by 24% at the maximum concentration (Fig. 3B). As shown in Fig. 1A, the 3 cell lines have different levels of proHB-EGF expression (NUGC-3 cells>5637 cells>BxPC-3 cells), which may explain the relative effect of Y-142 on proliferation. The 3 cell lines are derived from stomach, bladder, and pancreatic cancer tissues, which are known to overexpress HB-EGF [30]–[32]. HB-EGF has been reported to be upregulated in other cancer types such as breast and ovarian cancers and glioblastoma [33]–[35]; therefore, proHB-EGF may exert its functions in a broad range of cancer types.

In the spheroid culture, treatment with Y-142 increased the number of cells that diffused out of the spheroid core (Fig. 3A). The inhibition of proHB-EGF juxtacrine activity was detected even 3 d after the Y-142 treatment (Fig. 5), when cell diffusion was seen. We speculate that the proHB-EGF-mediated cell-cell contact is directly associated with its proHB-EGF juxtacrine activity. The results of a previous study using uc-proHB-EGF supported this speculation in that the results indicated a cytoprotective role of proHB-EGF by enhancing EGFR-mediated cell-cell contact and showed that caspase activation was inhibited by the proHB-EGF/EGFR/AKT pathway [28]. We also detected caspase activation and inhibited EGFR and AKT phosphorylation by Y-142 treatment in the spheroid culture (Figs. 4, 5A, and 5C). Our results may be derived from the inhibition of the cytoprotective role of proHB-EGF. We speculate that the anti-caspase function of proHB-EGF contributes to cancer cell survival by inhibiting apoptosis signals such as those activated in cells subjected to chemotherapeutic agents, radiotherapy, hypoxia, and immune cell-mediated cytotoxicity. A previous study reported that the anti-apoptotic protein BAG-1 mediates the proHB-EGF cell survival effect [36]. The BAG-1-mediated cell survival effect of proHB-EGF was noted in CHO cells, which are devoid of EGFR, suggesting that the proHB-EGF/BAG-1 pathway uses EGFR-independent cell survival signaling. proHB-EGF may use both the EGFR-dependent and EGFR-independent pathways to exert its cell survival effects. In addition, we noted the inhibition of ERK1/2 phosphorylation by Y-142 (Fig. 5B), suggesting that the cell proliferation signal from proHB-EGF is mediated through the ERK1/2 pathway. While measuring the juxtacrine activity of proHB-EGF in a 3D spheroid culture, we noted the inhibition of EGFR signaling by Y-142 (Fig. 5), suggesting that proHB-EGF exerts the anti-apoptotic and cell proliferation functions on neighboring cells through their EGFR pathway. In cases where cancer cells express both proHB-EGF and EGFR, the same cell may act as the donor and the acceptor of the cell survival and proliferation signals. Taken together, we predict that proHB-EGF efficiently induces tumor progression by directly promoting cancer cell proliferation and by inhibiting apoptosis. As shown in Fig. 6, Y-142 did not inhibit ectodomain shedding, leading to CTF production. Therefore, we concluded that the biological activity of Y-142 found here was not attributable to the inhibition of CTF production. Previous studies have indicated that both proHB-EGF and CTF translocate to the nucleus, thereby inducing cancer cell proliferation and invasion [37], [38]. If Y-142 possesses an inhibitory activity against proHB-EGF translocation, further analyses could identify the importance of the proHB-EGF translocation in cancer cell proliferation and invasion.

In this study, to investigate proHB-EGF functions in cancer cells, we used 2 anti-HB-EGF antibodies, Y-142 and Y-073, which had activities similar to those of sHB-EGF, but different specificities toward proHB-EGF (Figs. 1A and 2). Our previous study showed that the neutralizing activity of Y-142 is attributable to its recognition of amino acids in the EGF-like domain of HB-EGF [21]. The EGF-like domain is required for the binding of HB-EGF to EGFR. We therefore presume that the epitope of neutralizing antibody Y-073 should also be located in the EGF-like domain. Y-073, however, did not bind to proHB-EGF. These findings allow us to speculate that Y-073 may also recognize the newly exposed site in sHB-EGF after proHB-EGF ectodomain shedding. Consistent with our speculation, several antibodies to different molecules such as type II collagen, aggrecan, fibrin, and link protein have been reported to specifically recognize a newly exposed site resulting from a protein cleavage [39]. Epitope mapping studies will provide detailed information about Y-073 functionality.

In summary, we have shown that the endogenously expressed proHB-EGF acts as a cell proliferation and cell survival factor in cancer cells and that it may play an important role in cell proliferation and survival in tumor tissues.

## Supporting Information

Supporting Reference S1 **Reference 21.**
(PDF)
